# Targeting intestinal microecology: potential intervention strategies of traditional Chinese medicine for managing hypertension

**DOI:** 10.3389/fphar.2023.1171119

**Published:** 2023-05-31

**Authors:** Zhihua Yang, Shanshan Lin, Yangxi Liu, Zhihui Song, Zhao Ge, Yujian Fan, Lu Chen, Yingfei Bi, Zhiqiang Zhao, Xianliang Wang, Yi Wang, Jingyuan Mao

**Affiliations:** ^1^ First Teaching Hospital of Tianjin University of Traditional Chinese Medicine, National Clinical Research Center for Chinese Medicine Acupuncture and Moxibustion, Tianjin, China; ^2^ Institute of Traditional Chinese Medicine, Tianjin University of Traditional Chinese Medicine, Tianjin, China

**Keywords:** intestinal microecology, traditional Chinese medicine, hypertension, gut microbiota, gut barrier, short chain fatty acids

## Abstract

Hypertension has become one of the major public health problems in the world. At present, the pathogenesis of hypertension has still not been completely elucidated. In recent years, an increasing evidence shows that intestinal microecology is closely related to hypertension, which provides a new thinking for the prevention and treatment of hypertension. Traditional Chinese medicine (TCM) has unique advantages in the treatment of hypertension. Taking intestinal microecology as the target, it is possible to interpreting the scientific connotation of TCM prevention and treatment of hypertension by updating the treatment concept of hypertension, so as to improve the therapeutic effect. In our study, the clinical evidence for TCM treatment of hypertension was systematicly summarized. And the relationship among TCM, intestinal microecology and hypertension was analyzed. In addition, the methods by which TCM regulates intestinal microecology to prevent and treat hypertension were presented, to provide new research ideas for prevention and treatment of hypertension.

## 1 Introduction

The diagnostic criterion for hypertension is defined as systolic blood pressure (SBP) values of at least 140 mmHg and/or diastolic blood pressure (DBP) values of at least 90 mmHg (Williams et al., 2018). In 2015, the global prevalence of hypertension was estimated to be 1.13 billion based on office blood pressure (BP) (NCD Risk Factor Collaboration, 2017). The prevalence of hypertension will continue to rise as populations age, adopt more sedentary lifestyles, and gain weight. The number of people with hypertension is expected to rise by 15%–20% by 2025, approaching 1.5 billion (Kearney et al., 2005). The China hypertension survey in 2018 showed that the crude rate of adult hypertension in China reached 27.9%, showing a rising trend, and the awareness rate, treatment rate and control rate were 46.9%, 40.7%, and 15.3% respectively (Wang et al., 2018). Compared with the previous survey, although the overall improvement, its control rate is still relatively low. Hypertension is a major risk factor leading to stroke, myocardial infarction, heart failure and chronic kidney disease. And hypertension has been a major challenge to global health. Controlling BP is a key and effective measure to reduce the end-point events of heart, brain and kidney target organs (Mills et al., 2016; Williams et al., 2018; Unger et al., 2020). Meta-analyses of RCTs involving hundreds of thousands of patients have revealed that a 10 mmHg reduction in SBP or a 5 mmHg reduction in DBP is associated with a 20% reduction in all major cardiovascular events, a 10%–15% reduction in all-cause mortality, a 35% reduction in stroke, a 20% reduction in coronary events, and a 40% reduction in heart failure (Thomopoulos et al., 2014; Ettehad et al., 2016). High sodium intake, obesity, alcohol consumption, low potassium intake, physical inactivity and unhealthy diet are common risk factor for hypertension (Mills et al., 2020), changing lifestyle may be a effect strategy for controling BP level. At present, the commonly used antihypertensive drugs in clinical practice include beta-blockers, diuretics, angiotensin converting enzyme inhibitors, angiotensin II (Ang II) receptor antagonists, and calcium channel blockers (Laurent, 2017). Combined medication can significantly improve the rate of BP reaching the standard, but there are some other problems such as drug-related adverse reactions, application contraindications, drug resistance, and so on. How to further improve the prevention and treatment level of hypertension, delay the occurrence and development of hypertension, and improve the poor prognosis of hypertension is a major challenge at present and even in the future.

Traditional Chinese medicine (TCM) has a long history, and it has gradually formed a relatively complete system of theory, principle, prescription and medicine, which is widely used in the treatment of hypertension. In recent years, the advantages of multi-component, multi-target, multi-pathway, and overall comprehensive regulation of TCM in the treatment of hypertension have attracted much attention. Especially for some young patients and patients with primary hypertension, it is expected to reduce or even stop taking Western medicine (WM) and reduce adverse reactions through TCM syndrome differentiation and treatment. A recent randomized, multicenter, double-blind, parallel-group trials with 628 mild essential hypertension patients revealed that Songling Xuemaikang capsule, a Chinese herbal formula, was non-inferior to losartan in reducing office sitting DBP, sitting SBP, and 24-h ambulatory BP monitoring, as well as significantly improved hypertension symptom and TCM symptoms such as irritability, flushed face, and red eyes (Lai et al., 2022). A randomized, placebo-controlled trial including 251 patients with masked hypertension study found that gastrodia-uncaria granules could mildly reduce daytime and 24-h ambulatory BP and did not result in any adverse events (Zhang et al., 2020). Hypertension is the result of the combined action and superposition of multiple factors, especially genetic and environmental factors (Sekar et al., 2017). There are numerous pathological mechanisms involved in the occurrence and development of hypertension. The pathogenesis of hypertension has not been fully elucidated. Previous studies have shown that the antihypertensive mechanisms of TCM are related to blocking renin-angiotensin-aldosterone system, blocking calcium channels, protecting endothelial function and inhibiting vascular remodeling, improving insulin resistance, inhibiting sympathetic hyperactivity (Liu and Huang, 2016). In recent years, there is increasing evidence that intestinal microecological imbalance is closely related to hypertension and that maintaining intestinal microecological balance has an important role in BP regulation (Fandriks, 2017; Yang et al., 2023). Taking intestinal microecology as the starting point, this paper systematically summarized the clinical evidence of hypertension treatment by TCM, analyzed the relationship between intestinal microecology, TCM and hypertension, and expounded the possible intervention of TCM in hypertension through regulating intestinal microecology, improving intestinal barrier function, affecting the composition and abundance of gut microbiota, changing the metabolites of gut microbiota. This gives a stronger theoretical foundation for TCM prevention and treatment of hypertension. In the future, the improvement of intestinal microecology dysbiosis by TCM may become a new target for the prevention and treatment of hypertension.

## 2 TCM in the treatment of hypertension

### 2.1 Understanding of hypertension in TCM theory

There is no “hypertension” in ancient books of TCM, but there are some symptoms associated with hypertension. According to its clinical manifestations, hypertension can be classified into the categories of TCM diseases such as “Xuanyun” (vertigo), “Toutong” (headache), and “Zhongfeng” (stroke). TCM scholars believe that hypertension is related to emotional disorders, improper diet, chronic illness, overwork, aging, physical deficiency, and other factors. The *Expert Consensus on Diagnosis and Treatment of Hypertension with Traditional Chinese Medicine* (Society of Cardiovascular Diseases, 2019), published in 2019, divided hypertension into three TCM syndrome types (including the pattern of ascendant hyperactivity of liver yang, the pattern of phlegm and fluid retention, the pattern of kidney yin deficiency), and described in detail the diagnostic essentials and corresponding treatment methods of each TCM syndrome type, including prescription recommendation, adjustment plans of TCM composition, precautions, etc. Under the guidance of TCM theory based on the holistic concept and syndrome differentiation, TCM combined with conventional WM in the treatment of hypertension can flexibly apply individualized comprehensive diagnosis and treatment mode, and give full play to the advantages of synergistically lowering BP, enhancing the effect and reducing toxicity, improving symptoms and quality of life (Liu and Zhang, 2015).

### 2.2 Clinical evidence of TCM for hypertension

Clinical studies have shown that TCM combined with conventional WM in the treatment of hypertension has better efficacy and safety (Xinke et al., 2016). It can not only relieve clinical symptoms such as headache and vertigo, and improve the quality of life of patients, but also effectively and stably lower BP, which can provide an alternative for patients who cannot tolerate conventional WM. Moreover, it has multi-target and multi-pathway action characteristics and a protective effect on important organs (Wu and Dong, 2015). 38 representative clinical studies of TCM for hypertension are summarized in Supplementary Table S1, including 13 kinds of TCM decoction, 16 kinds of oral Chinese patent medicines (CPMs), and 9 kinds of TCM injection.

#### 2.2.1 TCM decoction for hypertension

##### 2.2.1.1 Clinical symptoms

1) Guipi decoction, Banxia Baizhu Tianma decoction, Bushen Huoxue decoction, Wendan decoction, Jianling decoction, Zhengan Xifeng decoction, Xiaochaihu decoction, and Tianma Gouteng decoction can improve the clinical efficacy, including the total clinical effective rate (Li and Wang, 2021; Yang et al., 2022; Wang et al., 2022; Ding et al., 2022; Liu, 2022; Qi, 2022; Wu and Zhong, 2022) the total effective rate of TCM syndromes (Yan et al., 2022) and the antihypertensive effective rate (Yan et al., 2022). 2) Banxia Baizhu Tianma decoction, Bushen Huoxue decoction, Chaihu Longgu Muli decoction, Wendan decoction, Jianling decoction, and Tianma Gouteng decoction can lower BP levels, including 24 h SBP, 24 h DBP, daytime SBP (DSBP), daytime DBP (DDBP), nighttime SBP (NSBP) and nighttime DBP (NDBP) (Li and Wang, 2021; Liu et al., 2021; Zhang et al., 2021; Zheng, 2021; Yang et al., 2022; Wang et al., 2022; Ding et al., 2022; Qi, 2022; Xia, 2022; Yan et al., 2022). 3) Zhengan Xifeng decoction reduced 24 h SBP variability (SBPV), and 24 h DBP variability (DBPV) (Liu, 2022). 4) Banxia Baizhu Tianma decoction, Chaihu Longgu Muli decoction, Wendan decoction, Jianling decoction, Tianma Gouteng decoction, Zhengan Xifeng decoction, and Xiaochaihu decoction can reduce TCM syndrome scores (Li and Wang, 2021; Zhang et al., 2021; Zheng, 2021; Yang et al., 2022; Wang et al., 2022; Liu, 2022; Qi, 2022; Wu and Zhong, 2022; Xia, 2022; Yan et al., 2022). It can also improve the TCM clinical symptoms, such as headache (Liu et al., 2021; Qi, 2022; Yan et al., 2022), dizziness (Liu et al., 2021; Zhang et al., 2021; Yang et al., 2022; Qi, 2022; Yan et al., 2022), chest tightness and shortness of breath (Yang et al., 2022), palpitation (Qi, 2022; Yan et al., 2022), insomnia (Qi, 2022; Yan et al., 2022), etc.

##### 2.2.1.2 Laboratory finding

1) Banxia Baizhu Tianma decoction, and Tianma Gouteng decoction can reduce the levels of aldosterone (ALD), Ang II, and plasma renin activity (Liu et al., 2021; Zheng, 2021). 2) Banxia Baizhu Tianma decoction and Wendan decoction can decrease endothelin-1 (ET-1) level (Li and Wang, 2021; Yang et al., 2022) and increase nitric oxide (NO) level (Li and Wang, 2021; Yang et al., 2022). 3) Banxia Baizhu Tianma decoction can also reduce whole blood high shear viscosity, whole blood low shear viscosity, and whole blood viscosity, and improve coagulation function (Li and Wang, 2021; Zhang et al., 2021). 4) Bushen Huoxue decoction can reduce serum hypersensitivity C-reactive protein (hs-CRP), interleukin-6 (IL-6), tumor necrosis factor α (TNF-α), and interleukin-8 (IL-8) levels (Ding et al., 2022). 5) Zhengan Xifeng decoction can reduce the level of homocysteine (Hcy) (Liu, 2022).

#### 2.2.2 Oral CPMs for hypertension

##### 2.2.2.1 Clinical symptoms

1) Fufang Danshen, Getong Tongluo capsule, Annao tablets, Polygoni Cuspidati Folium capsule, Qinggan Jiangya capsule, Songling Xuemaikang capsule, Gastrodin Tablets, Xinmaitong capsule, Yangxue Qingnao granules, Qiangli Dingxuan tablets, and Quanduzhong capsule can improve the clinical efficacy, including the total clinical effective rate (Yang et al., 2012; An, 2021; Ding and Gao, 2021; Yin and Yan, 2021; Wang et al., 2022; Chen et al., 2022; Deng, 2022; Jiang et al., 2022; Shi et al., 2022; Su et al., 2022) and antihypertensive effective rate (Zhao et al., 2021; Shi et al., 2022; Zhao and Zhao, 2022). 2) Fufang Danshen, Gastrodia-Uncaria granules, Songling Xuemaikang capsule, Annao tablets, Polygoni Cuspidati Folium capsule, Qinggan Jiangya capsule, Quanduzhong capsule, Shanhaidan granules, Gastrodin tablets, Xinmaitong capsule, Yangxue Qingnao granules, Xinkeshu tablets, Qiangli Dingxuan tablets, and Angong Jiangya pills can lower BP (Yang et al., 2012; Li and Wang, 2019; Zhang et al., 2020; An, 2021; Ding and Gao, 2021; Liang et al., 2021; Yin and Yan, 2021; Wang et al., 2022; Chen et al., 2022; Cui et al., 2022; Deng, 2022; Huang et al., 2022; Lai et al., 2022; Shi et al., 2022; Su et al., 2022; Zhao and Zhao, 2022), including 24 h SBP, 24 h DBP, 24 h standard deviation of SBP, 24 h standard deviation of DBP, 24 h mean pulse pressure, DSBP, DDBP, NSBP, NDBP, and pulse pressure, SBP load. Angong Jiangya pills can also improve the morning peak, valley peak ratio, and smoothing index of BP (Li and Wang, 2019). 3) Fufang Danshen and Qinggan Jiangya capsules can reduce pulse rate (Yang et al., 2012; Shi et al., 2022), and Shanhaidan granules can improve the standard deviation normal to the normal heartbeat in 24 h Holter electrocardiogram (Huang et al., 2022). 4) Annao tablets, Po lygoni Cuspidati Folium capsule, Qinggan Jiangya capsule, Qinggan Jiangya capsule, Shanhaidan granules, Songling Xuemaikang capsule, Xinmaitong capsule, and Angong Jiangya pills can reduce TCM syndrome scores (An, 2021; Ding and Gao, 2021; Wang et al., 2022; Chen et al., 2022; Huang et al., 2022; Shi et al., 2022) and improve TCM clinical symptoms such as headache (Ding and Gao, 2021; Liang et al., 2021; Chen et al., 2022), dizziness (Ding and Gao, 2021; Liang et al., 2021), irritability (Ding and Gao, 2021; Liang et al., 2021), fatigue, nausea, palpitation, and insomnia (Li and Wang, 2019; Liang et al., 2021). 5) Xinkeshu tablets can improve quality of life (Cui et al., 2022).

##### 2.2.2.2 Laboratory finding

1) Qingnao Jiangya tablets and Gastrodin tablets can reduce the levels of ALD, Ang Ⅰ, Ang Ⅱ, and the incidence of rheumatoid arthritis (Zhao and Zhao, 2022). 2) Polygoni Cuspidati Folium capsule, Qinggan Jiangya capsule, Qingnao Jiangya tablets, Xinmaitong capsule, and Yangxue Qingnao granules can significantly reduce the level of ET-1 (Ding and Gao, 2021; Wang et al., 2022; Su et al., 2022; Zhao and Zhao, 2022), von Willebrand factor (Zhao and Zhao, 2022), transforming growth factor-β1 (Zhao and Zhao, 2022), lipid peroxides, lipoprotein-related phospholipase A2 (Wang et al., 2022), vascular endothelial growth factor (VEGF) (Liang et al., 2021), and increase the level of NO (Ding and Gao, 2021; Wang et al., 2022; Su et al., 2022; Zhao and Zhao, 2022). 3) Polygoni Cuspidati Folium capsule can significantly reduce carotid and femoral pulse wave velocity and ankle-brachial pulse wave velocity (Ding and Gao, 2021). Xinmaitong capsule can reduce pulse wave velocity and improve the elasticity index of large arteries and small arteries (Wang et al., 2022). Yangxue Qingnao granules can reduce systemic circulation resistance, cardiac output, and cardiac index (Su et al., 2022). 4) Xinkeshu tablets can reduce the levels of TNF-α, hs-CRP, and IL-6 (Cui et al., 2022). 5) Qinggan Jiangya capsule, Songling Xuemaikang capsule, and Yangxue Qingnao granules can reduce serum Hcy level (An, 2021; Liang et al., 2021; Su et al., 2022). 6) Xinkeshu tablets can regulate blood lipids, including reducing triglyceride (TG), total cholesterol (TC), and low-density lipoprotein cholesterol (LDL-C) (Cui et al., 2022). 7) Qingnao Jiangya tablets can reduce the levels of humoral immune indexes IgA, IgM, IgG, and C3 (Zhao and Zhao, 2022).

#### 2.2.3 TCM injection for hypertension

##### 2.2.3.1 Clinical symptoms

1) Puerarin injection, Mai-Luo-Ning injection, Sofren injection, Sodium Tanshinone ⅡA Sulfonate injection, and Danhong injection can improve the total clinical effective rate (Liang, 2020; Mai et al., 2020; Song et al., 2020; Yao et al., 2021; Zhang and Wang, 2022). 2) Puerarin injection, Danshen injection, Gastrodin injection, Mai-Luo-Ning injection, Compound Danshen injection, Huangqi injection, Sofren injection, and Danhong injection can regulate BP, including SBP, DBP, 24 h standard deviation of DBP, 24 h standard deviation of SBP, the standard deviation of DSBP, the standard deviation of NSBP (Mai et al., 2020; Song et al., 2020; He and Zhang, 2021; Xie, 2021; Yao et al., 2021; Lv, 2022; Zhang et al., 2022; Zhang and Wang, 2022). 3) Puerarin injection can also reduce TCM syndrome scores, including improving headache, chest tightness, palpitation, and numbness of hands and feet (Zhang and Wang, 2022).

##### 2.2.3.2 Laboratory finding

1) Compound Danshen injection, Sofren injection, and Danhong injection can decrease the level of ET-1 and increase the level of NO (Mai et al., 2020; Song et al., 2020; Yao et al., 2021). Sofren injection can also decrease the level of thromboxin 2 and increase the level of 6-keto-prostaglandin F1α (Song et al., 2020). 2) Danshen injection can reduce the serum levels of IL-6, C-reactive protein, and TNF-α (Zhang et al., 2022). Mai-Luo-Ning injection can reduce IL-18 level (Yao et al., 2021). 3) Compound Danshen injection can reduce blood viscosity (He and Zhang, 2021). Danhong injection can reduce the levels of TC, TG, and LDL-C, and increase the levels of high-density lipoprotein cholesterol (HDL-C) (Mai et al., 2020). 4) Mai-Luo-Ning injection can decrease the levels of Hcy and D-dimer (Yao et al., 2021). 5) Compound Danshen injection can decrease the level of D-dimer and reduce urinary protein content (He and Zhang, 2021), and Huangqi injection can reduce serum creatinine and 24 h urinary protein (Lv, 2022). 6) Sofren injection can decrease left ventricular end-diastolic diameter (LVEDD) and left ventricular end-systolic diameter (LVESD) (Song et al., 2020).

In recent years, integrated Chinese and WM in the treatment of hypertension has been widely used in clinical practice and has achieved good clinical efficacy. Many clinical studies and analyses proved that integrated Chinese and WM therapy were superior to those using WM alone for hypertension. A systematic review and meta-analysis of randomized controlled trials (RCTs) including 3,823 patients with postmenopausal hypertension revealed that compared with antihypertensive drugs treatment alone, traditional herbal medicine (THM) with antihypertensive drugs treatment could further significantly reduce SBP and DBP, relieve postmenopausal symptoms, ameliorate physical and physical symptoms, improve the level of estradiol, reduce the levels of follicle-stimulating hormone, luteinizing hormone, and testosterone (Xiong et al., 2019c). Another similar systematic review and meta-analysis of RCTs involving 1,460 hypertensive patients demonstrated that THM adjuvant to antihypertensive drugs was more effective than antihypertensive drugs treatment alone in lowering BP, ameliorating depression, decreasing the levels of serum total cholesterol, triglycerides, and low-density lipoprotein cholesterol, raising the level of serum high-density lipoprotein cholesterol, and reducing the levels of Hcy and CRP (Xiong et al., 2019b).

To sum up, clinical evidence indicates that TCM is beneficial for treating hypertension in 1) reducing BP level, including 24 h SBP, 24 h DBP, 24 h SBPV, 24 h DBPV, DSBP, DDBP, NSBP, and NDBP; 2) relieving the typical clinical symptoms of headache, dizziness, chest tightness, and palpitation; improving TCM syndrome scores; improving quality of life; 3) inhibiting body inflammatory reaction, including decreasing the levels of IL-6, hs-CRP, TNF-α, D-dimer, and Ang-II; improving blood lipids, including reducing the levels of TG, TC, and LDL-C and increasing the level of HDL-C; improving vascular endothelial functions, which include decreasing the levels of ET-1 and Hcy, increasing the levels of NO and VEGF.

## 3 The relationship between TCM and intestinal microecology

**FIGURE 1:**
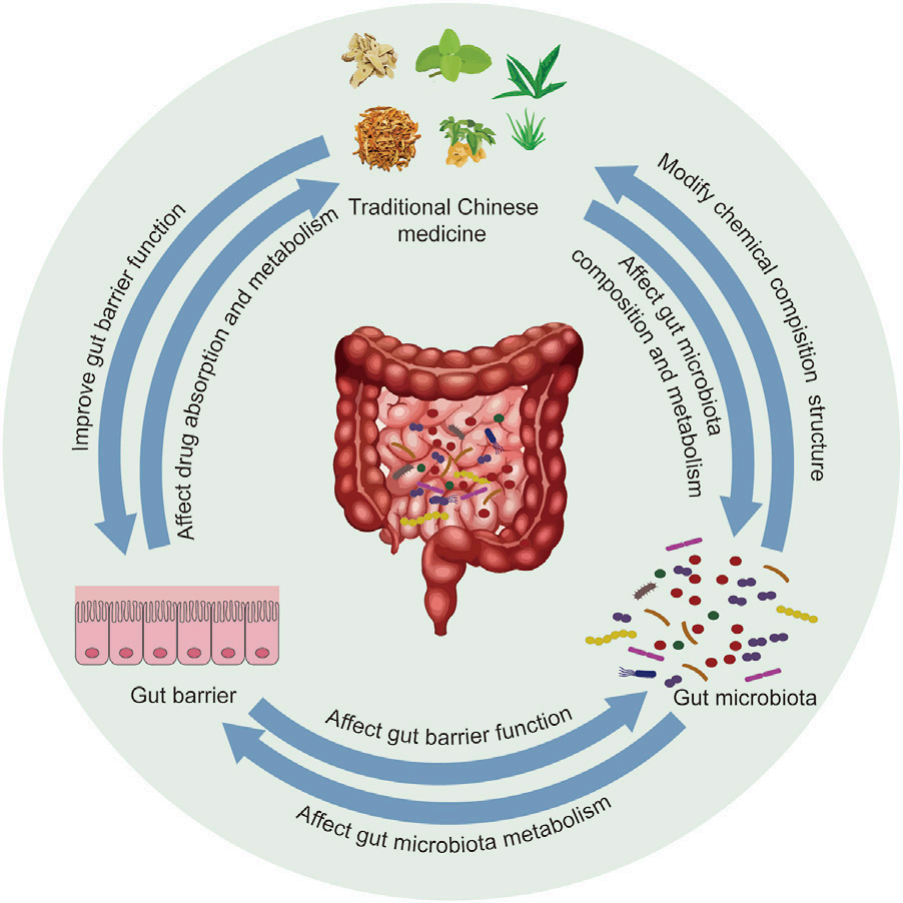
The interaction between TCM and intestinal microecology.

The gut microbiota of the human body and its living intestinal environment constitute intestinal microecology, which is the main and most complex microecology of the human body, and the gut microbiota occupies the core position of intestinal microecology. According to the relationship between gut microbiota and host, gut microbiota can be divided into beneficial bacteria, neutral bacteria and harmful bacteria (Yang et al., 2022c; Feng et al., 2023). The composition of the gut microbiota is dynamic, which has some individual differences, and is related to the environment, age, diet, disease, drugs, and genetics (Human Microbiome Project, 2012). When the homeostasis of intestinal microecology is disrupted, beneficial bacteria will decline and harmful bacteria will increase, the gut barrier will be damaged, and the function of the immune system will be disrupted, which will trigger a variety of diseases and threatens human health (Yang et al., 2022d). There is an interaction between TCM and intestinal microecology (Figure 1). TCM is mainly oral decoction, the intestinal tract is an important metabolic place of oral TCM in the body, and the interaction between TCM and gut microbiota is often the key to its efficacy. The interaction between TCM and gut microbiota in the body is mainly in two aspects. One is that TCM can affect the composition and metabolism of gut microbiota, which is mainly reflected in that TCM can promote the proliferation of beneficial bacteria and inhibit the excessive growth of harmful bacteria, to restore the intestinal microecological balance under disease conditions and play a role in the treatment of diseases. On the other hand, under the action of gut microbiota, TCM can generate metabolites with stronger pharmacological activity, generate more toxic metabolites, or transform toxic drugs into low-toxic or non-toxic metabolites, to play a role (Xu et al., 2017; Feng et al., 2019). TCM can not only affect the composition and structure of gut microbiota but also promote intestinal mucosal repair, protect the intestinal mucosal barrier, and influence the location of gut microbiota.

## 4 Correlation between intestinal microecology and hypertension

### 4.1 Intestinal barrier and hypertension

Both clinical and animal studies on hypertension are associated with intestinal barrier dysfunction. Intestinal barrier dysfunction, especially in individuals with long-term conditions, poor BP management, cardiac and renal problems, and the use of numerous antihypertensive drugs are all associated with hypertension (Li et al., 2020). Kim et al. (2018) discovered that hypertensive patients had significant increases in plasma of intestinal fatty acid binding protein, lipopolysaccharide (LPS), and augmented gut-targeting proinflammatory T helper 17 (Th17) cells, indicating increased intestinal inflammation and permeability. Li et al. (2020) study found that high BP patients had higher rates of elevated diamine oxidase and LPS than normotensive subjects, indicating that hypertension was linked to serious intestinal barriers impairment, such as small intestinal epithelial injury and endotoxin translocation. The spontaneously hypertensive rats (SHRs) model exhibits clear changes in intestinal pathology, including shorter villi and goblet cells, increased gut permeability, and inflammation, which suggests that hypertension caused intestinal barrier dysfunction (Jaworska et al., 2017; Santisteban et al., 2017).

### 4.2 Gut microbiota and hypertension

Gut microbiota refers to the trillions of symbiotic microorganisms distributed in the intestine in a certain proportion. Appropriate gut microbiota and their metabolites are essential to maintain the homeostasis of the body’s internal environment, while the imbalance of gut microbiota can lead to a variety of diseases (Tang et al., 2019). Both animal and clinical studies on hypertension showed decreased gut microbiota richness and diversity (Yang et al., 2015; Li et al., 2017; Yan et al., 2017). A clinical study using metagenomic to analyze fecal samples from patients with hypertension and healthy controls found the gut microbiota dysbiosis characterized by decreased gut microbiota diversity, increased pathogenic bacteria, and decreased short-chain fatty acids (SCFAs)-producing bacteria in hypertensive patients (Yan et al., 2017). Sun et al. (2019) conducted the first population-based cohort study on the relationship between gut microbiota and hypertension, and found that gut microbiota diversity was inversely associated with hypertension. Palmu et al. (2020) investigated the link between the gut microbiota and BP in a sample of 6,953 Finns study participants and found that individuals with hypertension demonstrate changes in several microbiota genera, with most of these genera belonging to the *Firmicutes phylum*. In the SHRs and chronic Ang II infusion rat model, Yang et al. (2015) found a significant decrease in microbial richness, diversity, and evenness, as well as an increase in the *Firmicutes/Bacteroidetes* (F/B) ratio.

Hypertension is associated with gut microbiota dysbiosis, both in animal and human hypertension (Yang et al., 2015). The decrease in gut microbiota abundance, diversity and the increase of the ratio of F/B can increase the risk of hypertension (Hills et al., 2019). Some studies have shown that gut microbiota can directly affect the BP of the host. After the transplantation of fecal microbiota from spontaneously hypertensive stroke-prone rats was transferred to normotensive rats by fecal microbiota transplantation (FMT) technique, it was found that the BP of control rats also increased significantly (Adnan et al., 2017). After the transplantation of feces from hypertensive patients to germ-free mice using FMT, the BP of germ-free mice also increased significantly and the structure of gut microbiota also changed (Li et al., 2017). In addition, clinical trials have found that the BP of hypertensive patients decreased after probiotic consumption. A meta-analysis of 14 RCTs involving 702 hypertensive patients showed that probiotic fermented milk could significantly reduce systolic and diastolic BP in hypertensive patients (Dong et al., 2013). On the other hand, hypertension can cause a variety of complications including chronic kidney disease, which could affect intestinal protein fermentation, change gut intraluminal pH, promote intestinal urea excretion, and eventually cause gut microbiota disorder (Felizardo et al., 2016; Yang et al., 2018a). It can be seen that hypertension and gut microbiota disorder can form a vicious circle.

### 4.3 Gut microbiota metabolites and hypertension

**FIGURE 2:**
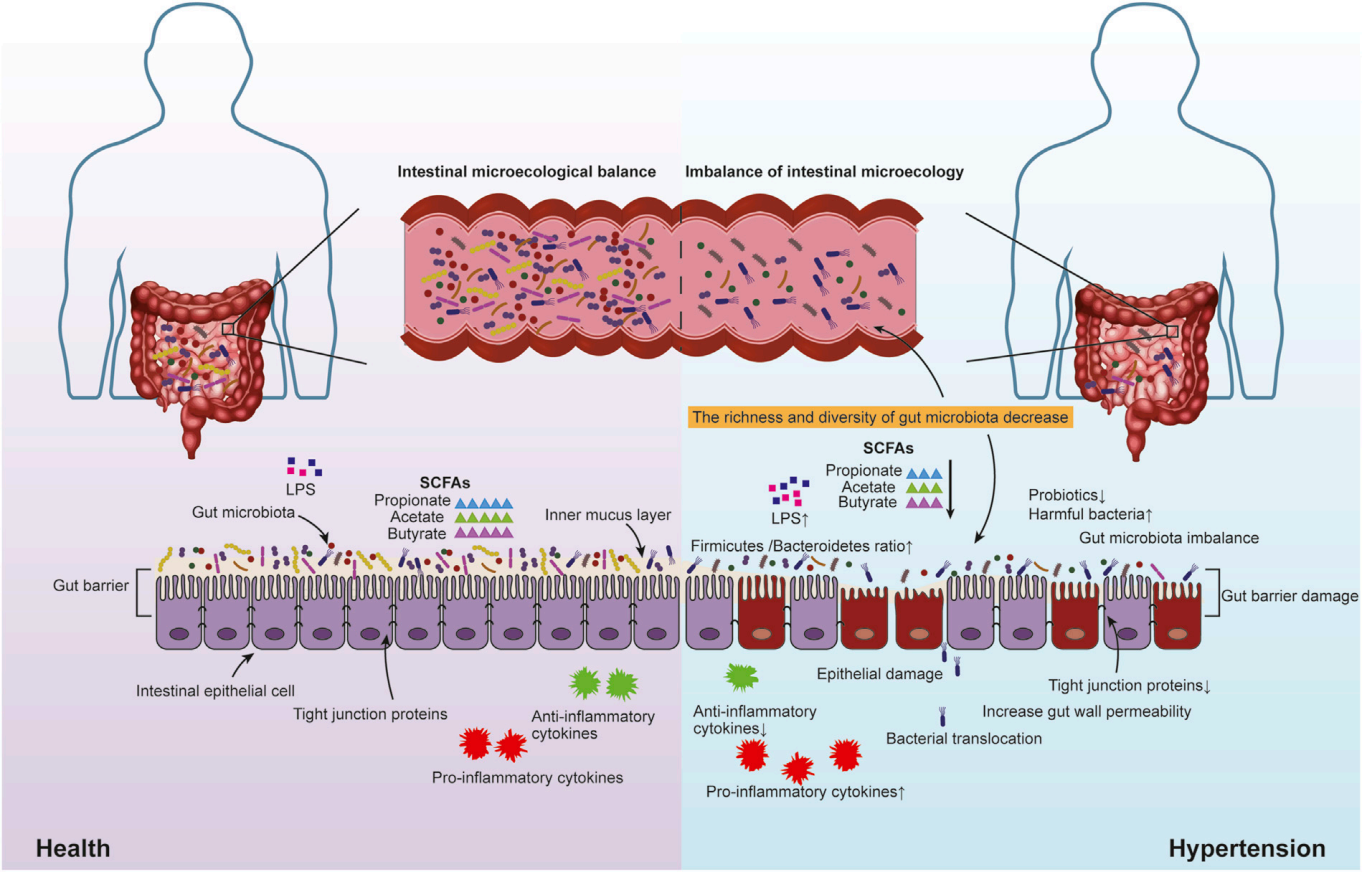
In a healthy state, the intestinal microecology of the human body is in dynamic balance. Intestinal microecology disorders in hypertensive patients include decreased abundance and diversity of gut microbiota, increased harmful bacteria, decreased beneficial bacteria, abnormal metabolites of gut microbiota, decreased tight junction protein, intestinal barrier dysfunction, increased intestinal permeability and so on.

Gut microbiota takes part in host metabolism and produces a variety of substances that affect BP, such as SCFAs, trimethylamine-N-oxide (TMAO), bile acids (BAs), corticosterone and hydrogen sulfide (H_2_S). SCFAs are an important metabolite of gut microbiota, which can reduce BP, regulate immunity and protect the heart and kidney. SCFAs are mainly produced by colon bacteria ferment polysaccharides (fibers) that cannot be digested by the human body, including butyrate, acetate and propionate, etc. These three metabolites account for 80% of SCFAs produced by gut microbiota. They can affect BP by activating G protein-coupled receptor (GPCR) (Brinks and Eckhart, 2010; Vieira-Rocha et al., 2020), modulating immune-inflammatory response (Bartolomaeus et al., 2019), renin-angiotensin-aldosterone system (RAAS) (Marques et al., 2017), autonomic nerve (Yang et al., 2018b; Onyszkiewicz et al., 2019). Studies have found that the number and types of butyrate-producing gut microbiota are reduced in hypertensive people. Treatment of AngⅡ-induced hypertensive mice with butyrate could make the increased mean arterial pressure approach the safe range of BP, restore the damaged intestinal barrier, and correct the imbalance of gut microbiota (Kim et al., 2018). TMAO is another important metabolite produced by gut microbiota. *In vivo* animal experiments, it was found that the increase of circulating TMAO can increase the retention of sodium and water by increasing plasma osmolality, stimulating the release of plasma vasopressin (PAVP), up-regulating the expression of aquaporin-2 (AQP-2) in the apical membrane of main cells of the renal collecting duct. That is, BP is raised through the “TMAO-AVP-AQP-2 axis” (Liu et al., 2019). TMAO can also enhance the low-dose AngⅡ-mediated pressor effect and make the pressor effect last longer (Ufnal et al., 2014). BAs are the main component of bile, and their synthesis and metabolism are completed with the participation of gut microbiota. BAs are considered to be an important component of digestion and absorption, as well as to regulate metabolism by activating intestinal, liver and peripheral receptors (Wahlstrom et al., 2016). It is suggested that BAs may affect the occurrence and development of hypertension by regulating vascular endothelial function (Fiorucci et al., 2017), affecting kidney function (Herman-Edelstein et al., 2018), and production of TMAO (Verhaar et al., 2020; Duttaroy, 2021). Based on gut microbiota imbalance, various pathological factors will affect BAs homeostasis, resulting in the occurrence and development of diseases including hypertension. Under physiological conditions, the content of corticosterone produced by the intestine is very low, which increases significantly when intestinal inflammation occurs. Corticosterone can activate mineralocorticoid receptors, leading to water and sodium retention and elevated BP (Yan et al., 2020). Like mammalian cells and tissues, gut microbiota can also produce H_2_S gas, which can participate in several physiological processes (including smooth muscle relaxation, oxidative regulation and inflammation). H_2_S may reduce BP by reducing the synthesis and release of renin. Especially when the body has too much H_2_S, it can inhibit RASS (Weber et al., 2016; Huc et al., 2018). In addition, H_2_S may also reduce BP by dilating peripheral blood vessels and reducing heart rate (Hartley et al., 2016). LPS is a component of Gram-negative bacteria such as *Escherichia coli*. In animal experiments, LPS can be used to induce vascular dysfunction (Battson et al., 2018). The integrity of the intestinal barrier and the expression of tight junction proteins in patients with hypertension are damaged, and LPS in the intestine can reach various tissues and organs in the body through blood circulation, triggering the body’s inflammatory response and aggravating hypertension (Kim et al., 2018; Sun et al., 2018). Figure 2.

## 5 Ways of TCM to treat hypertension by regulating intestinal microecology

**FIGURE 3:**
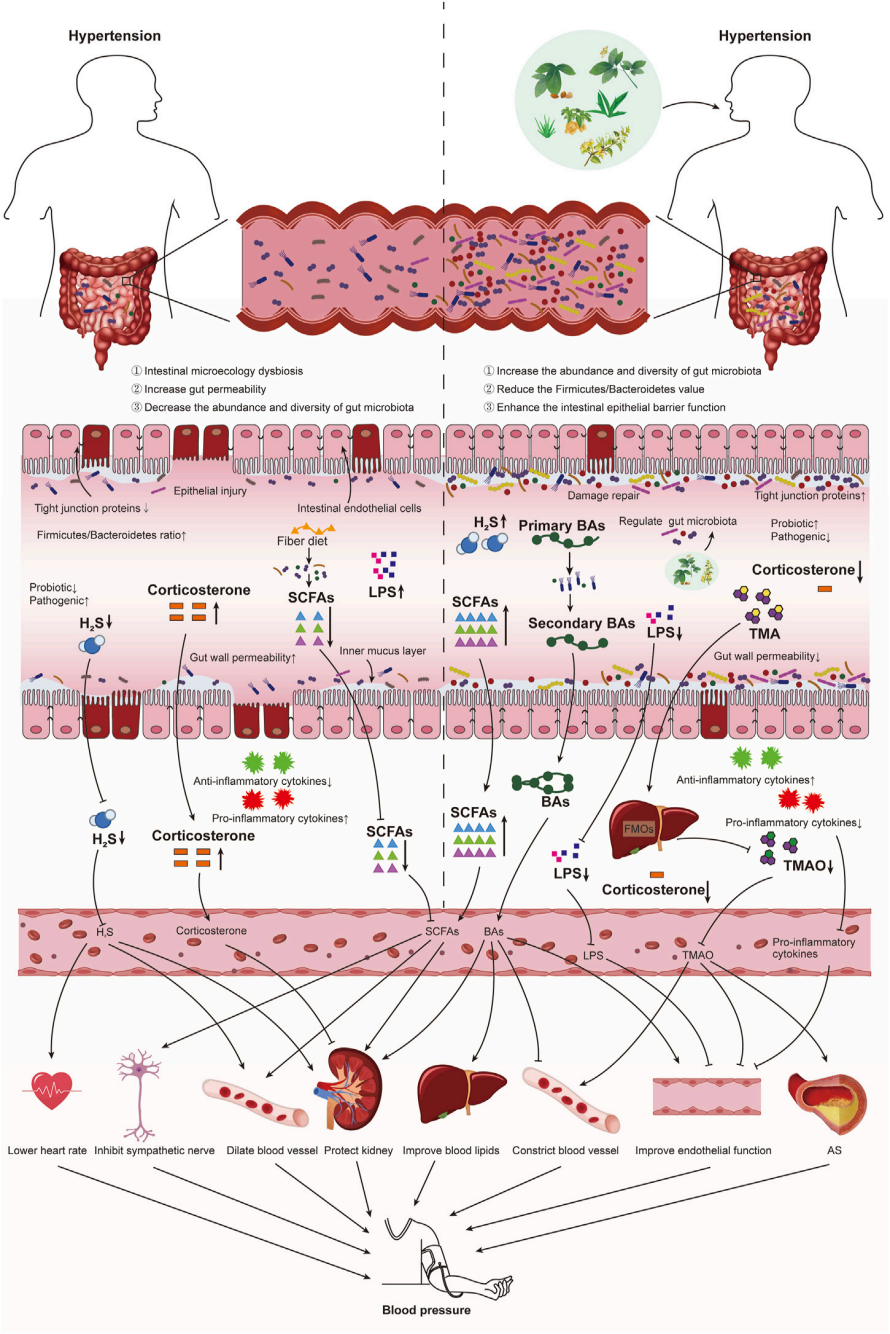
Ways of TCM to treat hypertension by regulating intestinal microecology. Hypertension patients with intestinal microecology disorder, intestinal barrier dysfunction, increased intestinal permeability, and gut microbiota imbalance, resulting in abnormal production of gut microbiota metabolites (SCFAs, TMAO, BAs, H_2_S, and LPS). Metabolites produced by the gut microbiota cross the intestinal barrier into the blood vessels, and they reach the whole body through blood circulation to affect organ function and then affect BP. On the one hand, TCM improves BP by improving intestinal barrier function, increasing intestinal tight junction protein and reducing intestinal permeability. On the other hand, it regulates BP by improving gut microbiota disorders and regulating gut microbiota metabolites (SCFAs, TMAO, BAs, H_2_S, and LPS).

Hypertension’s development and occurrence are highly correlated with intestinal microecology. Therefore, investigating intestinal microecology-based intervention methods may offer new approaches to treating hypertension. Currently, antibiotics, supplementation of probiotics, fecal microbiota transplantation, diet and exercise, antihypertensive drugs, and natural medicines are the main ways to manage intestinal microecology (Yang et al., 2023). In recent years, the research of TCM on the prevention and treatment of hypertension by regulating intestinal microecology has been increasing. Studies have shown that TCM can treat hypertension by adjusting the ratio of probiotics to pathogenic bacteria, restoring the diversity of gut microbiota, improving intestinal barrier function, and regulating the metabolites of gut microbiota (Figure 3). Basic and clinical study of TCM for hypertension by regulating intestinal microecology as shown in Table 1.

**TABLE 1:** Basic and clinical study of TCM for hypertension by regulating intestinal microecology.

No	Botanical drugs/metabolites	Composition or source	Model	Duration of treatment	Doses (mg/kg)	Test method	Main findings	Implicated microbiota	References
1	Zhengan Xifeng decoction	Achyranthes bidentata Blume [Amaranthaceae; achyranthis bidentatae radix], Pisaster ochraceus (Brandt, 1835) [Asteriidae; ochra], Fossilia Ossis Mastodi (Baill.) [Fabaceae; Os Draconis], *Crassostrea gigas* (Thunberg) [Ostreidae; Ostreae Testa], *Chinemys reevesii* (Gray) [Geoemydidae; Testudinis Carapax et Plastrum], Paeonia lactiflora Pall. [Paeoniaceae; paeoniae radix alba], Scrophularia ningpoensis Hemsl. [Scrophulariaceae; scrophulariae radix], Asparagus cochinchinensis (Lour.) Merr. [Asparagaceae; asparagi radix], Melia azedarach L. [Meliaceae; meliae cortex], Hordeum vulgare L. [Poaceae; hordei fructus germinatus], Artemisia capillaris Thunb. [Asteraceae; artemisiae scopariae herba], Glycyrrhiza glabra L. [Fabaceae; glycyrrhizae radix et rhizoma praeparata cum melle]	SHRs	8 weeks	15, 30, 60	16S rDAN	① Decrease serum diamine oxidase and D-lactate levels ② Improve the expression of zonula occluden-1, Occludin mRNA in the colon ③ Protect the intestinal mucosal barrier	① Change the proportion of bacteria producing acetic acid, propionic acid, and butyric acid in SHRs	Xu et al. (2020)
			SHRs	8 weeks	15,000, 30,000, 60,000	16S rDAN			Yu et al. (2019)
2	Baicalin	Scutellaria baicalensis Georgi [Lamiaceae; scutellariae radix]	Ang II-Induced hypertensive mice	4 Weeks	12, 60, 300	16S rDAN	① Decrease SBP and DBP ② Protect intestinal barrier function and reduce intestinal permeability ③ Attenuate the necrotic and ulcerative intestinal lesions and impairment of the mechanical intestinal barrier④ Decrease the decreased the serum levels of hs-CRP, IL-6, and IL-1β⑤ Mitigate intestinal hyperpermeability and systemic inflammatory response	① Increase the microbial production of SCFA	Li et al. (2022)
			SHRs	6 Weeks	100	16S rDAN			Wu et al. (2019)
3	Huangqin-Huaihua	Scutellaria baicalensis Georgi [Lamiaceae; scutellariae radix], Styphnolobium japonicum (L.) Schott [Fabaceae; sophorae flos]	SHRs	14 Weeks	900, 1,800	16S rRAN	① Lower BP	① Improve gut microbiota dysbiosis, decreased the F/B ratio	Guan. (2020)
							② Reduce blood creatinine and urea nitrogen levels		
							③ Inhibit renal fibrosis and improve renal function		
							④ Increase the expression of intestinal tight junction protein and repair the damage of intestinal barrier		
4	Cheqianzi crude polysaccharide	Plantago asiatica L. [Plantaginaceae; plantaginis semen]	Hypertensive patients	2 Weeks	Unreported	RT-PCR	① Decrease SBP and DBP	① Increase the abundance of *Bacteroides* and Bifidobacterium	Li et al. (2019)
							② Protect intestinal barrier function and reduce intestinal permeability		
							③ Attenuate the necrotic and ulcerative intestinal lesions and impairment of the mechanical intestinal barrier		
							④ Decrease the decreased the serum levels of hs-CRP		
5	Rhynchophylline	Uncaria hirsuta Havil. [Rubiaceae; uncariae ramulus cum uncis]	SHRs	6 Weeks	2.5, 5, 10	16S rDAN	① Ameliorate BP	① At the Phylum level, the abundance of Firmicutes decreased significantly as well as the abundance of Bacteroidetes increased significantly	Zhang et al. (2023)
								② At the Genus level, the abundance of Oscillospira, Ruminococcus decreased significantly as well as the abundance of Prevotella increased significantly	
6	Quercetin	In the flowers, leaves, or fruits of plants	SHRs	8 Weeks	50	16S rDAN	① Lower SBP	① Increase the abundance and diversity of intestinal flora	Zhou et al. (2020)
							② Improved the degree of myocardial fibrosis	② Decrease the F/B ratio	
7	Soyasaponin Bb	Soybeans and some legumes	High-salt-induced hypertensive mice	3 Weeks	1.51	16S rDAN	① Lower SBP and DBP	① Reduce the relative abundance of harmful bacteria, increase the relative abundance of probiotics	Zhang et al. (2019)
8	Gegen extract	Pueraria montana var. lobata (Willd.) Maesen and S.M.Almeida ex Sanjappa & Predeep [Fabaceae; puerariae lobatae radix]	High-salt-induced hypertensive mice	8 Weeks	3.75	16S rDAN	① Ameliorate BP	① Increase the abundance of *Clostridium*	Li et al. (2020b)
9	Huangqi-Danshen	Astragalus mongholicus Bunge [Fabaceae; astragali radix praeparata cum mell], Salvia miltiorrhiza Bunge [Lamiaceae; salviae miltiorrhizae radix et rhizoma]	SHRs	4 Weeks	3,230, 6,450	16S rDAN	① Ameliorate BP	① Decrease the F/B ratio	Han et al. (2019)
								② Improve the abundance of probiotics such as *Lactobacillus* and Bifidobacterium	
10	Duzhong-Cijili	Eucommia ulmoides Oliv. [Eucommiaceae; eucommiae cortex], Tribulus terrestris L. [Zygophyllaceae; tribuli fructus]	SHRs	8 Weeks	2,760, 5,503, 11,060	16S rDAN	① Ameliorate BP	① Increase the abundance and diversity of intestinal flora	Qi et al. (2019)
							② Reduce serum NT-proBNP, CRP, and Ang II levels	② Decrease the relative abundance of Actinobacterica	
11	Qinggan Yishen Qufeng decoction	Conioselinum anthriscoides ‘Chuanxiong’ [Apiaceae; chuanxiong rhizoma], Leonurus japonicus Houtt. [Lamiaceae; leonuri fructus], Taxillus chinensis (DC.) Danser [Loranthaceae; taxilli herba], Bupleurum chinense DC. [Apiaceae; bupleuri radix], Coptis chinensis Franch. [Ranunculaceae; coptidis rhizoma], Achyranthes bidentata Blume [Amaranthaceae; achyranthis bidentatae radix], Hansenia weberbaueriana (Fedde ex H.Wolff) Pimenov & Kljuykov [Apiaceae; notopterygii rhizoma et radix], Saposhnikovia divaricata (Turcz. ex Ledeb.) Schischk. [Apiaceae; saposhnikoviae radix], Polygonatum sibiricum Redouté [Asparagaceae; polygonati rhizoma], Scutellaria baicalensis Georgi [Lamiaceae; scutellariae radix], Haliotis Linnaeus, 1758. [Haliotidae; Concha Haliotidis], Prunella vulgaris L. [Lamiaceae; prunellae spica]	SHRs	14 Weeks	Unreported	16S rDAN	① Decrease SBP② Promote the secretion of acetic acid and butyric acid in the intestine in hypertensive rats③ Improve intestinal pathological damage	① Increase the abundance and diversity of intestinal flora② Promote the growth of intestinal Bifidobacteria, downregulate the abundance of Proteobacteria	Tang et al. (2021)
			Ang II-Induced hypertensive mice	4 Weeks	Unreported	16S rDAN			Zhen et al. (2021)
12	Xiaochaihu decoction	Bupleurum chinense DC. [Apiaceae; bupleuri radix], Scutellaria baicalensis Georgi [Lamiaceae; scutellariae radix], Codonopsis pilosula (Franch.) Nannf. [Campanulaceae; codonopsis radix], Glycyrrhiza glabra L. [Fabaceae; glycyrrhizae radix et rhizoma praeparata cum melle], Pinellia ternata (Thunb.) Makino [Araceae; pinelliae rhizoma], Zingiber officinale Roscoe [Zingiberaceae; zingiberis rhizoma recens], Ziziphus jujuba Mill. [Rhamnaceae; jujubae fructus]	Hypertensive patients	2 Months	Unreported	Unreported	① Decrease SBP and DBP	① Increase the abundance of *Bacteroides*, *Lactobacillus*, and Bifidobacter, decrease the abundance of *Enterococcus*, yeast, and *Enterobacter*	Wu et al. (2022)
							② Reduce serum TG, TC, and LDL-C levels, Improve serum HDL-C level		
							③ Improve clinical efficacy		
							④ Reduce serum IL-6, TNF-cyLDL-C levels"bi		
13	Huanglian Jiedu decoction	Coptis chinensis Franch. [Ranunculaceae; coptidis rhizoma], Scutellaria baicalensis Georgi [Lamiaceae; scutellariae radix], Phellodendron chinense C.K.Schneid. [Rutaceae; phellodendri chinensis cortex], Gardenia jasminoides J.Ellis [Rubiaceae; gardeniae fructus]	SHRs	6 Weeks	27,000	16S rDAN	① Decrease BP	① Reduce the relative abundance of Firmicutes, increase the relative abundance of probiotic *Lactobacillus* ② Increase the diversity of intestinal flora	Ma et al. (2020)
14	Sanhuang Xiexin decoction	Rheum tanguticum (Maxim. ex Regel) Balf. [Polygonaceae; rhei radix et rhizoma], Scutellaria baicalensis Georgi [Lamiaceae; scutellariae radix], Coptis chinensis Franch. [Ranunculaceae; coptidis rhizoma]	SHRs	1 Week	1,300, 2,600, 6,000	16S rRAN	① Lower BP	① Increase the amount of *Lactobacillus* ② Alter the percentage compositions of Corynebacterium, *Lactobacillus*, and Turicibacter bacteria	Wu et al. (2020)
15	Bushen Hemai granules	Astragalus mongholicus Bunge [Fabaceae; astragali radix praeparata cum mell], Polygonatum sibiricum Redouté [Asparagaceae; polygonati rhizoma], Taxillus chinensis (DC.) Danser [Loranthaceae; taxilli herba], Epimedium brevicornu Maxim. [Berberidaceae; epimedii folium], Eucommia ulmoides Oliv. [Eucommiaceae; eucommiae cortex], Ligustrum lucidum W.T.Aiton [Oleaceae; cera chinensis], Achyranthes bidentata Blume [Amaranthaceae; achyranthis bidentatae radix], Alisma plantago-aquatica subsp. Orientale (Sam.) Sam. [Alismataceae; alismatis rhizoma], Conioselinum anthriscoides ‘Chuanxiong’ [Apiaceae; chuanxiong rhizoma], Angelica sinensis (Oliv.) Diels [Apiaceae; angelicae sinensis radix], Pheretima guillelmi (Michaelsen) [Megascolecidae; Lumbricus]	SHRs	8 Weeks	25,000	16S rDAN	① Decrease SBP, DBP, mean arterial pressure, and pressure pulse	① Decrease the F/B ratio	Liu et al. (2019a)
								② Increase the diversity of intestinal flora	
			Hypertensive patients	8 Weeks	Unreported	16S rDAN	① Lower BP	① Decrease the F/B ratio	Zhang. (2021)
							② Reduce clinical efficiency and TCM syndrome score	② Increase the abundance and diversity of intestinal flora	
16	Chai-Gui Decoction	Bupleurum chinense DC. [Apiaceae; bupleuri radix], Scutellaria baicalensis Georgi [Lamiaceae; scutellariae radix], Zingiber officinale Roscoe [Zingiberaceae; zingiberis rhizoma recens], Pinellia ternata (Thunb.) Makino [Araceae; pinelliae rhizoma], Glehnia littoralis (A.Gray) F.Schmidt ex Miq. [Apiaceae, glehniae radix], Angelica sinensis (Oliv.) Diels [Apiaceae; angelicae sinensis radix], Conioselinum anthriscoides ‘Chuanxiong’ [Apiaceae; chuanxiong rhizoma], Paeonia lactiflora Pall. [Paeoniaceae; paeoniae radix alba], Wolfiporia cocos (Schw.) Ryv. and Cilbn. [Polyporaceae; poria], Atractylodes macrocephala Koidz. [Asteraceae; atractylodis macrocephalae rhizoma], Alisma plantago-aquatica subsp. Orientale (Sam.) Sam. [Alismataceae; alismatis rhizoma]	SHRs	4 Weeks	3,000, 5,000, 15,000	16S rRAN	① Lower BP and reduce the aortic wall thickness in SHR rats	① Increase the abundance and diversity of intestinal flora	Zhu et al. (2023)
							② Increase the percentage of angiotensin 1–7 (Ang 1–7), decrease the percentage of	② Decrease the F/B ratio	
							Ang II, and decrease the Ang II/Ang 1of ib_zh		
17	Qingxuan granules	Astragalus mongholicus Bunge [Fabaceae; astragali radix praeparata cum mell], Neolitsea cassia (L.) Kosterm. [Lauraceae; cinnamomi cortex], Gastrodia elata Blume [Orchidaceae; gastrodiae rhizoma], Piper kadsura (Choisy) Ohwi [Piperaceae; piperis kadsurae caulis], Conioselinum anthriscoides ‘Chuanxiong’ [Apiaceae; chuanxiong rhizoma], Wolfiporia cocos (Schw.) Ryv. and Cilbn. [Polyporaceae; poria], Atractylodes macrocephala Koidz. [Asteraceae; atractylodis macrocephalae rhizoma], Rhodiola crenulata (Hook.f. and Thomson) H.Ohba [Crassulaceae; rhodiolae crenulatae radix et rhizoma], Ziziphus jujuba Mill. [Rhamnaceae; jujubae fructus]	Hypertensive patients	8 Weeks	Unreported	16S rRAN	① Decrease BP	① Decrease the F/B ratio	Mei. (2022)
							② Reduce clinical efficiency and TCM syndrome score	② Increase the abundance and diversity of intestinal flora	
							③ Reduce the levels of TC and LDL-C		
18	Xianxiong Chengqi decoction	Trichosanthes kirilowii Maxim. [Cucurbitaceae; trichosanthis fructus], Pinellia ternata (Thunb.) Makino [Araceae; pinelliae rhizoma], Rheum tanguticum (Maxim. ex Regel) Balf. [Polygonaceae; rhei radix et rhizoma], Potassium nitrate J.R. Glauber [Potassium salts; natrii sulfas], Citrus tassium nitrate[Rutaceae; aurantii fructus immaturus], Coptis chinensis Franch. [Ranunculaceae; coptidis rhizoma]	Hypertensive patients	4 Weeks	Unreported	16S rDAN	① Lower BP	① Decrease the F/B ratio	Zhang. (2022)
							② Improve clinical efficiency and TCM syndrome	② Increase the abundance and diversity of intestinal flora	

TCM, traditional Chinese medicine; BP, blood pressure; DBP, diastolic blood pressure; SBP, systolic blood pressure; SHRs, spontaneously hypertensive rats; hs-CRP, hypersensitivity C-reactive protein; IL-6, interleukin-6; IL-1β, interleukin-1β; F/B Firmicutes/Bacteroidetes; Ang II, angiotensin II; TC, total cholesterol; LDL-C, low-density lipoprotein cholesterol.

### 5.1 TCM treats hypertension by improving gut barrier function

Hypertensive patients are often accompanied by impaired gut barriers (Zhou et al., 2018). Gut barrier damage will affect the growth of probiotics, resulting in dysbacteriosis, which in turn affects the formation of the gut barrier and energy supply, thus forming a vicious circle. At the same time, after the gut barrier is damaged, its permeability increases, and pathogens and inflammatory substances are more likely to pass through the gut barrier, further aggravating intestinal damage and accelerating the progression of the disease. Relevant studies have shown that TCM can reduce intestinal mucosal permeability by increasing intestinal transepithelial resistance and tight junctions, thereby enhancing the intestinal epithelial barrier function, inhibiting the entry of intestinal pathogenic bacteria and enterotoxin LPS into the body, thereby reducing the body’s inflammation and lower BP. Xiong et al. (2019) found that Qinggan Yishen Qufeng compound could improve the intestinal permeability of hypertensive rats, increase the expression of intestinal tight junction protein, and reduce the intestinal damage caused by hypertensive lesions. Yu et al. (2019) found that Zhengan Xifeng decoction could effectively reduce the F/B value of hypertensive mice, while promoting the production of SCFAs, thereby maintaining the ecological balance of the intestinal tract and protecting the integrity of the intestinal mechanical barrier (Gu and Zhang, 2016). Baicalin is the main flavonoid component of *Scutellariae Radix*. Li et al. (2022) and Wu et al. (2019) reported baicalin could exert protective effects on intestinal integrity under hypertensive conditions, which treatment could alleviate the necrotic and ulcerative intestinal lesions, impairment of the mechanical intestinal barrier, mitigate systemic inflammatory response and intestinal hyperpermeability, reduce decreased the serum levels of hs-CRP, IL-1β, and IL-6 in the SHRs. Huangqin-Huaihua can increase the expression of ZO-1 and Occulidin protein in SHRs colon tissue, repair the damaged intestinal structure, and improve intestinal barrier function (Guan, 2020).

### 5.2 TCM treats hypertension by regulating gut microbiota

#### 5.2.1 TCM active ingredients

Li et al. (2019) found that the number of *Bifidobacterium* and *Bacteroides* in the gut microbiota of hypertension patients increased significantly after Cheqianzi crude polysaccharide treatment, and the BP decreased significantly. Zhang et al. (2023) found that rhynchophylline has a definite antihypertensive effect, and can improve gut microbiota disorder to a certain extent, improve the abundance of intestinal beneficial bacteria and optimize the composition of gut microbiota in SHRs. Quercetin and resveratrol are polyphenolic organic metabolites. Studies have shown that polyphenolic organic metabolites can regulate the structure of gut microbiota by inhibiting harmful bacteria and promoting the growth of beneficial bacteria (Ozdal et al., 2016), and can be further metabolized and absorbed by gut microbiota such as *Bifidobacterium*, *Lactobacillus* and *Bacteroides* (Cardona et al., 2013). After treating SHRs with quercetin, Zhou et al. (2020) found that the BP of rats was significantly decreased, the F/B ratio was decreased, and the abundance and diversity of gut microbiota were improved, suggesting that quercetin could reduce BP by regulating gut microbiota. Kim et al. (2018) transplanted fecal microbiota from healthy mice fed with resveratrol into AngⅡ-induced hypertensive mice and found that the SBP of hypertensive mice decreased. These results indicate that resveratrol is sufficient to reduce the BP of AngⅡ-induced hypertensive mice and it reduces the BP of mice by regulating gut microbiota. Zhang et al. (2019) found that soyasaponin Bb could significantly alleviate the increase of BP caused by a high-salt diet in mice, and reduce the relative abundance of some intestinal bacteria that increased after a high-salt diet, and also increase the relative abundance of some intestinal bacteria that decreased during high-salt diet.

#### 5.2.2 Botanical drug

Li et al. (2020) confirmed that Gegen extract can improve the BP increase induced by a high-salt diet in mice by reducing the abundance of *Spirillaceae* and *Erythrobacillus* and increasing the abundance of *Clostridium* and *Vibrio*. Han et al. (2019) used Huangqi-Danshen to treat SHRs for 28 days and found that the BP of SHRs decreased, the F/B ratio increased, and the abundance and diversity of gut microbiota increased. And the relative abundance of *Lactobacillus*, *Bifidobacterium* and other probiotics related to BP reduction increased. *Akkerman-sia muciniphila*, *Lactobacillus intestinalis* and *Lactobacillus reuteri* were found to be associated with BP regulation. Qi et al. (2019) used Duzhong-Cijili to intervene SHRs with different concentrations and doses. The results showed that Duzhong-Cijili could reduce SHRs BP and inflammation levels, increase gut microbiota abundance and SCFAs levels, and reduce fecal acetic acid, propionic acid, butyric acid, isobutyric acid, valerate and isovalerate levels. It was suggested that Duzhong-Cijili decreased aged SHRs BP, which was closely related to the improvement of gut microbiota composition. Guan (2020) intervened SHRs with Huangqin-Huaihua botanical drug pair, and used a 16S rRNA high-throughput sequencing technique to analyze the intestinal microbes of rats in each group. The results showed that Huangqin-Huaihua botanical drug pair could improve the diversity and abundance of gut microbiota in SHRs, reduce the F/B ratio, and improve the disorder of gut microbiota.

#### 5.2.3 Chinese medicine compounds

Tang et al. (2021) found that Qinggan Yishen Qufeng decoction could increase the relative abundance of *Bifidobacteriales* and *Bifidobacterium* in gut microbiota, and decrease the abundance of *Proteobacteria*. It also can promote the production of SCFAs such as acetic acid and butyric acid in the intestine, thus maintaining intestinal homeostasis and reducing SHRs BP. Zhen et al. (2021) further found that Qinggan Yishen Qufeng decoction can inhibit Ang II-induced hypertension. Its mechanism may be through improving the destruction of the intestinal structural barrier and regulating the imbalance of gut microbiota and fecal metabolites. Wu et al. (2022) explored the effect of Xiaochaihu decoction combined with irbesartan on gut microbiota and lipid metabolism in hypertension patients. It was found that compared with the irbesartan group, Xiaochaihu decoction combined with the irbesartan group could increase the number of *Bacteroides*, *Lactobacillus*, *Bifidobacterium* and other probiotics in hypertension patients, improve lipid metabolism, reduce BP and regulate gut microbiota disorder. Zhengan Xifeng decoction can change the proportion of acetic acid-producing bacteria, propionic acid-producing bacteria and butyric acid-producing bacteria in SHRs, repair the damaged intestinal mucosa, and reduce the release of D-lactate, diamine oxidase and other inflammatory factors into the blood circulation, thus reducing BP (Xu et al., 2020). Ma et al. (2020) found that Huanglian Jiedu decoction can improve the diversity of gut microbiota and the relative abundance of *Lactobacillus* in SHR, and reduce the relative abundance of *Firmicutes*. It is inferred that Huanglian Jiedu decoction may reduce BP by regulating the structure of gut microbiota, promoting the growth of beneficial gut microbiota and inhibiting the growth of harmful gut microbiota. Wu et al. (2020) compared the gut microbiota of SHRs before and after administration, and found that the percentage of *Corynebacterium*, *Lactobacillus* and *Pseudomonas aeruginosa* changed significantly, and the number of *Lactobacillus* increased obviously. Relevant reports have shown that *Lactobacillus* is closely related to hypertension, and it can produce effective vasodilator NO and antihypertensive neurotransmitters (Yang et al., 2015). These results indicate that Sanhuang Xiexin decoction can increase the number of beneficial bacteria such as *Lactobacillus*, effectively dilate blood vessels, protect vascular endothelial cells, and further improve BP. In addition, Bushen Hemai granules (Liu et al., 2019; Zhang, 2021), Chai-Gui decoction (Zhu et al., 2023), Qingxuan granules (Mei, 2022), and Xianxiong Chengqi decoction (Zhang, 2022) all have the significant effect in lowering decrease BP, which might be related to increasing the diversity of gut microbiota and decreased the F/B ratio.

### 5.3 TCM treats hypertension by regulating gut microbiota metabolites

SCFAs are an important class of gut microbiota metabolites, which can directly dilate blood vessels and reduce BP (Miyamoto et al., 2016). SCFAs produced by probiotics in the human body can also be absorbed into the circulatory system through the gastrointestinal tract to affect BP (Jose and Raj, 2015). Zhenggan Xifeng decoction, a TCM formula, can effectively reduce the BP and heart rates of SHR by maintaining the integrity of the gut mechanistic barrier and increasing the percentage of microbes producing SCFAs (Yang et al., 2020). In addition, Qi et al. (2019) found that *Eucommia ulmoides-Tribuli Fructus* reduced BP in SHR by altering the level and composition of SCFAs in the gut by modulating gut microbiota composition and diversity. Studies have shown that some natural small-molecule metabolites, such as berberine (Shi et al., 2018) and Guneulsterone (Gautam et al., 2019), can act on the gut microbiota-TMA-TMAO metabolic pathway, thereby reducing the level of TMAO in peripheral blood. Berberine contained in TCMs such as *Coptidis Rhizoma* can reduce the expression of FMO_3_ and the level of serum TMAO in the liver of ApoE^−/−^ mice with high-fat diet-induced atherosclerosis (AS) (Shi et al., 2018). Studies have shown that resveratrol can regulate BAs by down-regulating the expression of the hepatic enterofarnesoid X-like receptor-fibroblast growth factor 15 axis, and ultimately exert an anti-atherosclerotic effect (Chen et al., 2016). Zhou et al. (2020) found that quercetin might reduce LPS production and immune inflammatory response by improving gut microbiota, thereby improving vascular dysfunction and vascular remodeling, and reducing BP. In conclusion, TCM can reduce BP by regulating the metabolites of gut microbiota.

## 6 Summary and prospect

Intestinal microecology is the largest micro-ecosystem in the human body, which participates in a variety of important physiological functions and is closely related to a variety of diseases. The rapid development of intestinal microecology provides a new perspective for revealing the occurrence regularity of diseases and the effect mechanism of drugs. Intestinal microecology plays an important role in the occurrence and development of cardiovascular diseases, and the regulation of intestinal microecology is a potential new target for the treatment of hypertension (Yang et al., 2023). Antihypertensive therapy targeting the intestinal microecology is a very promising treatment mode, attaching importance to the intestinal microecology as target of hypertension research, broadening the research and treatment of hypertension, for the future development of antihypertensive microbial preparation to provide the related reference, to solve the existing medical mode of hypertension treatment difficulties such as resistant hypertension has great inspiration. The study on the pathway of gut microbiota involvement in the formation of hypertension is not only aimed at exploring what causes it but also provides a large number of new targets for the clinical treatment of hypertension, such as the artificial production of flora metabolism that can regulate BP, and selection of different types of antihypertensive drugs according to different intestinal metabolic characteristics. In addition, taking intestinal microecology as the target, paying attention to the effect of TCM on intestinal microecology, and interpreting the scientific connotation of TCM in the prevention and treatment of hypertension may renew the treatment concept of hypertension and improve the therapeutic effect of hypertension.

Currently, more and more research confirmed that hypertension can be treated by regulating the gut microbiota with TCM, but most of the experiments are confined to animal studies, lacking the validation of the clinical trials, and TCM composition complex, the single active ingredient is difficult to a large number of purification, the problem of low biological availability. In addition, most experiments only stay in observing the antihypertensive effect of drugs and the level of corresponding structure changes in gut microbiota. The specific mechanism and target of TCM intervention on intestinal microecology to lower BP have not been studied in depth. At present, the research on the relationship between intestinal microecology and hypertension is still in the initial stage, and the research methods are not mature enough. In addition, the relationship between intestinal microecology and the human body is very complicated, and it is easily affected by diet, exercise, other drugs, psychological factors and other factors when studying the relationship between intestinal microecology and hypertension. Therefore, future research should also focus on the study of the internal mechanism related to flora.

There are some limitations in this study: 1) The quality of clinical evidence on the treatment of hypertension by TCM included in this study is generally low. At present, most clinical studies use TCM as an admixture of Western medicine in the treatment of hypertension, and the clinical sample size is small, the follow-up period is short, and the design of clinical studies is not standardized, so the reliability of clinical efficacy is still to be discussed. 2) At present, there are relatively few studies on the treatment of hypertension by TCM through the regulation of intestinal microecology. The quality of evidence is generally low, and there is a lack of in-depth analysis and discussion. For example, due to the complexity of TCM compound components, which components regulate intestinal flora and the specific mechanisms, and whether intervening these mechanisms can improve intestinal microecology, clinical symptoms, and prognosis of hypertensive patients are worth exploring. Given the above scientific problems, we suggest that future studies should focus on the following aspects: 1) To carry out high-quality studies with large samples, multi-centers, rigorous design, strict implementation, and standard reporting to provide reliable and accurate evidence support for the effectiveness and safety of TCM treatment of hypertension. 2) To further explore the relationship between intestinal microecology and hypertension and the deeper molecular mechanism of TCM prevention and control of hypertension by regulating intestinal microecology. 3) In addition, 16S rDNA, 16S rRNA, enterobacterial repetitive intergenic consensus-PCR, ultrahigh performance liquid chromatography quadrupole-time of flight-mass spectrometry (UPLC-QTOF/MS) method, genomics, transcriptomics, and proteomics techniques can be used to reveal the active ingredients, molecular mechanisms and intervention targets of TCM in regulating intestinal flora to prevent hypertension.

## Figures and Tables

**Table:** 

**TCM**	traditional Chinese medicine
**BP**	blood pressure
**DBP**	diastolic blood pressure
**SBP**	systolic blood pressure
**WM**	Western medicine
**CPMs**	Chinese patent medicines
**DSBP**	daytime SBP
**DDBP**	daytime DBP
**NSBP**	nighttime SBP
**NDBP**	nighttime DBP
**SBPV**	SBP variability
**DBPV**	DBP variability
**ALD**	aldosterone
**Ang II**	angiotensin Ⅱ
**ET-1**	endothelin-1
**NO**	nitric oxide
**hs-CRP**	hypersensitivity C-reactive protein
**IL-6**	interleukin-6
**TNF-α**	tumor necrosis factor α
**IL-8**	interleukin-8
**Hcy**	homocysteine
**VEGF**	vascular endothelial growth factor
**TG**	triglyceride
**TC**	total cholesterol
**LDL-C**	low-density lipoprotein cholesterol
**LVEDD**	left ventricular end-diastolic diameter
**LVESD**	left ventricular end-systolic diameter
**RCTs**	randomized controlled trials
**LPS**	lipopolysaccharide
**Th17**	T helper 17
**SHRs**	spontaneously hypertensive rats
**SCFAs**	short-chain fatty acids
**FMT**	fecal microbiota transplantation
**F/B**	Firmicutes/Bacteroidetes
**TMAO**	trimethylamine-N-oxide
**BAs**	bile acids
**H_2_S**	hydrogen sulfide
**GPCR**	G protein-coupled receptor
**AS**	atherosclerosis
**RAAS**	renin-angiotensin-aldosterone system
**PAVP**	plasma vasopressin
**AQP-2**	aquaporin-2
